# Association between preoperative pan-esophageal pressurization and peristalsis recovery after per-oral endoscopic myotomy in achalasia

**DOI:** 10.1093/gastro/goag081

**Published:** 2026-08-04

**Authors:** Dianxuan Jiang, Ruoxi Zhang, Peiwen Dong, Shimin Chen, Wei Zhao, Songfeng Chen, Niandi Tan, Mengyu Zhang, Qianjun Zhuang, Xiaoqing Li, Qiong Wang, Nina Zhang, Yinglian Xiao

**Affiliations:** Department of Gastroenterology, The First Affiliated Hospital of Sun Yat-sen University, Guangzhou, Guangdong 510080, P. R. China; Department of Gastroenterology, Peking Union Medical College Hospital, Chinese Academy of Medical Sciences and Peking Union Medical College, Beijing 100730, P. R. China; Health Management Center, The Third People’s Hospital of Chengdu, The Affiliated Hospital of Southwest Jiaotong University, Chengdu, Sichuan 610000, P. R. China; Department of Gastroenterology, The First Affiliated Hospital of Sun Yat-sen University, Guangzhou, Guangdong 510080, P. R. China; Department of Gastroenterology, The Affiliated Drum Tower Hospital, Nanjing University Medical School, Nanjing, Jiangsu 210008, P. R. China; Department of Gastroenterology, The First Affiliated Hospital of Sun Yat-sen University, Guangzhou, Guangdong 510080, P. R. China; Department of Gastroenterology, The First Affiliated Hospital of Sun Yat-sen University, Guangzhou, Guangdong 510080, P. R. China; Department of Gastroenterology, The First Affiliated Hospital of Sun Yat-sen University, Guangzhou, Guangdong 510080, P. R. China; Department of Gastroenterology, The First Affiliated Hospital of Sun Yat-sen University, Guangzhou, Guangdong 510080, P. R. China; Department of Gastroenterology, Peking Union Medical College Hospital, Chinese Academy of Medical Sciences and Peking Union Medical College, Beijing 100730, P. R. China; Health Management Center, The Third People’s Hospital of Chengdu, The Affiliated Hospital of Southwest Jiaotong University, Chengdu, Sichuan 610000, P. R. China; Department of Gastroenterology, The Affiliated Drum Tower Hospital, Nanjing University Medical School, Nanjing, Jiangsu 210008, P. R. China; Department of Gastroenterology, The First Affiliated Hospital of Sun Yat-sen University, Guangzhou, Guangdong 510080, P. R. China

**Keywords:** achalasia, per-oral endoscopic myotomy, high-resolution manometry, peristalsis

## Abstract

**Background:**

Per-oral endoscopic myotomy (POEM) has become the mainstream treatment for achalasia. Previous studies have found that some patients with achalasia might experience partial recovery of esophageal peristalsis after POEM. However, the clinical significance of this recovery and the associated factors remain to be further elucidated.

**Methods:**

This was a multicenter retrospective cohort study conducted by four academic hospitals in China. Patients diagnosed with achalasia who underwent POEM between 2020 and 2025 were included. Demographics, Eckardt score, gastroesophageal reflux disease questionnaire (GERDQ) score, presence of esophagitis, high-resolution manometry parameters, and myotomy length were collected. Normal, weak, and fragmented contractions were defined as partial recovery of esophageal peristalsis.

**Results:**

Partial recovery of esophageal peristalsis was observed in 32.8% (95 of 290) of patients with achalasia. A significantly higher preoperative proportion of pan-esophageal pressurization was found in the recovery group compared with the non-recovery group [median (interquartile range), 80.0% (30.0%, 100.0%) vs 30.0% (0.0%, 90.0%), *P *< 0.001] and this proportion positively correlated with the degree of peristalsis recovery (*r* = 0.247, *P *< 0.001). Patients with peristalsis recovery had significantly lower GERDQ scores and reduced incidence of dysphagia symptoms. Multivariate analysis further confirmed that the preoperative proportion of pan-esophageal pressurization was an independent predictor of peristalsis recovery (odds ratio = 1.02, *P *= 0.007).

**Conclusions:**

A higher preoperative proportion of pan-esophageal pressurization was independently associated with postoperative recovery of esophageal peristalsis in patients with achalasia. Peristalsis recovery was related to superior symptom relief. Preoperative assessment of pan-esophageal pressurization might help guide individualized postoperative follow-up and patient counseling.

## Introduction

Achalasia is a primary esophageal motility disorder characterized by impaired relaxation of the lower esophageal sphincter (LES) and the absence of normal peristalsis in the esophageal body [1]. Patients typically present with dysphagia and regurgitation, which can progress to severe complications such as severe malnutrition, aspiration pneumonia, and malignant tumors due to chronic food retention in the esophagus. The worldwide annual incidence and prevalence of achalasia are estimated to range from 0.09 to 1.41 and from 6.83 to 16.90 per 100,000 persons, respectively [2]. High-resolution manometry (HRM) is the gold-standard diagnostic tool and, under the Chicago Classification version 4.0, achalasia is classified into three subtypes [3]. While all subtypes share an elevated integrated relaxation pressure (IRP), they differ in their esophageal peristalsis manometric features: type I is characterized by aperistalsis and absent pressurization, type II by pan-esophageal pressurization, and type III by premature contractions.

The primary pathogenesis of achalasia involves the loss of inhibitory neurons at the LES [4]. However, the exact etiology of this neuronal degeneration remains unclear. As a result, a radical cure for achalasia is still lacking. Currently, per-oral endoscopic myotomy (POEM) has become the mainstream treatment, given its minimal invasiveness and proven safety and efficacy in large cohort studies [5]. However, as regards POEM as a palliative treatment, ∼20% of patients with achalasia experience clinical recurrence (Eckardt score > 3) during the 2-year follow-up period after POEM [6]. Additionally, the disruption of the normal LES anatomy by POEM predisposes patients to gastroesophageal reflux disease (GERD), which is the most frequent delayed adverse event [7]. A meta-analysis of 1,542 patients reported post-POEM reflux symptoms in 19% of cases, abnormal acid exposure in 39% of cases, and esophagitis in 29.4% of cases [8].

Notably, although all three subtypes of achalasia lack normal peristalsis preoperatively, some patients may experience partial recovery of esophageal peristalsis following POEM after the relief of LES obstruction [9, 10]. However, studies investigating post-POEM peristalsis recovery remain limited and its clinical significance, underlying mechanisms, and contributing factors have yet to be fully elucidated.

This study aimed to investigate the clinical relevance of esophageal peristalsis recovery and to identify factors associated with its restoration in patients with achalasia after POEM.

## Methods

### Study subject enrollment

This was a multicenter retrospective cohort study conducted by four academic hospitals in China (the First Affiliated Hospital of Sun Yat-sen University, Guangzhou, Guangdong; the Affiliated Drum Tower Hospital of Nanjing University Medical School, Nanjing, Jiangsu; Peking Union Medical College Hospital, Beijing; and the Third People’s Hospital of Chengdu, Chengdu, Sichuan). Patients diagnosed with achalasia who underwent POEM between 2020 and 2025 were initially screened. Criteria for inclusion required preoperative endoscopy, upper gastrointestinal contrast radiography, and HRM, as well as follow-up endoscopy and HRM at 6 months post-POEM. Patients who could not be definitively diagnosed with achalasia based on HRM according to the Chicago Classification version 4.0 were excluded from the study [3].

Baseline data, including age, gender, body mass index (BMI), duration of symptoms, and Eckardt score, were collected. During the POEM procedure, the myotomy length was documented. At 6 months post-POEM, symptom questionnaire scores [Eckardt and gastroesophageal reflux disease questionnaire (GERDQ)], the incidence of typical GERD symptoms (heartburn and regurgitation), dysphagia, and esophagitis were also recorded. The presence of reflux esophagitis was assessed through endoscopy and graded according to the Los Angeles (LA) classification [11].

### HRM

HRM was performed by using a 4.2-mm outer-diameter solid-state manometric catheter with 36 circumferential sensors spaced at 1-cm intervals (Medtronic Inc, Minneapolis, MN, USA). The catheter was calibrated from 0 to 300 mmHg before being transnasally positioned. The protocol began with a 30-second baseline recording to allow patient adaptation, followed by 10 5-mL liquid swallows and 3 multiple rapid swallows performed in the supine position. After a subsequent 30-second baseline recording, five additional 5-mL liquid swallows were administered in the upright position. All manometric data were independently analysed by two investigators, who were blinded to the clinical information, according to the Chicago Classification version 4.0 [3].

**Figure 1:**
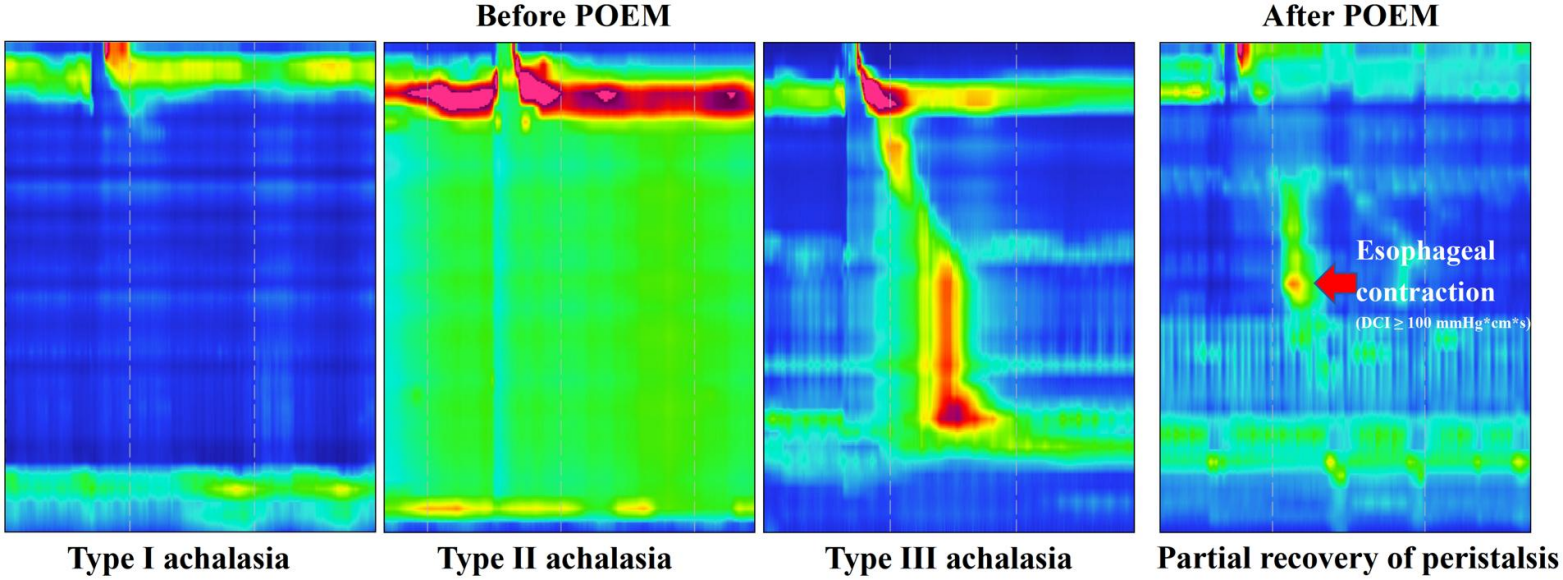
Esophageal pressure topography studies before and after POEM. POEM = peroral endoscopic myotomy; DCI = distal contractile integral.

Metrics evaluating LES function, including IRP and LES resting pressure, were measured both pre- and post-POEM. Parameters that assess esophageal motility patterns, such as the percentage of swallows with pan-esophageal pressurization, and premature, normal, weak, and fragmented contractions during 10 supine swallows were also recorded. Pan-esophageal pressurization was identified by using a 30-mmHg isobaric contour tool and defined according to the Chicago Classification version 4.0 as pressurization extending from the upper esophageal sphincter to the esophagogastric junction [3]. The preoperative proportion of pan-esophageal pressurization was calculated as the percentage of swallows demonstrating pan-esophageal pressurization among the 10 supine liquid swallows. Partial recovery of esophageal peristalsis was defined by the presence of normal, weak, or fragmented contractions during these swallows (Fig. 1). The degree of peristalsis recovery was quantified by using the total proportion of normal, weak, and fragmented contractions.

### POEM

All POEM procedures were performed in a standardized manner by experienced endoscopists under general anesthesia with endotracheal intubation, as described previously [12]. A myotomy length of > 12 cm was defined as a long myotomy [13].

### Outcomes

The primary outcome was partial recovery of esophageal peristalsis at 6 months after POEM. Secondary outcomes included the degree of peristaltic recovery, postoperative HRM parameters, GERDQ score, Eckardt score, dysphagia symptoms, GERD symptoms, and reflux esophagitis. Postoperative symptom occurrence was defined as the presence of heartburn, regurgitation, or dysphagia [defined as a visual analogue scale (VAS) score of > 0] during the follow-up.

### Statistical analysis

Continuous variables were reported as median (interquartile range) and compared by using the Mann–Whitney *U* test. Categorical variables were presented as number (percentage) and compared by using the chi-squared test. The statistically significant difference was set at *P* < 0.05. All the statistical analyses were performed by using SPSS Statistics version 22 (IBM, Armonk, NY, USA).

## Results

A total of 290 patients were enrolled, consisting of 140 males and 150 females, with a median age of 42.0 years. The demographic and clinical characteristics of the subjects are shown in Table 1. According to the Chicago Classification version 4.0, 98 patients were diagnosed with type I achalasia, 185 with type II, and 7 with type III. Postoperatively, partial recovery of esophageal peristalsis was observed in 95 (32.8%) of the patients. Among these patients, only six achieved normal peristalsis, whereas the remaining patients were classified as having ineffective esophageal motility according to the Chicago Classification version 4.0.

**Table 1:** Clinical characteristics of 290 patients with achalasia.

Variables	*n *= 290
Male	140 (48.3)
Age, years	42.0 (30.0, 54.0)
BMI, kg/m^2^	20.5 (18.3, 22.8)
Duration of symptoms, months	36.0 (18.7, 84.0)
Preoperative achalasia subtypes	
Type I	98 (33.8)
Type II	185 (63.8)
Type III	7 (2.4)
Partial recovery of peristalsis	95 (32.8)

Continuous variables are shown as median (interquartile range) and categorical variables are presented as number (percentage).

### Association between partial recovery of esophageal peristalsis after POEM and preoperative pan-esophageal pressurization

Patients were categorized into two groups based on their postoperative peristalsis recovery: those with partial recovery (*n *= 95) and those without (*n *= 195) (Table 2). No significant differences were found in preoperative characteristics, including gender, age, BMI, disease duration, Eckardt score, LES resting pressure, and IRP, as well as myotomy length during POEM and the proportion of patients undergoing long myotomy (all *P *> 0.05). Notably, the majority (78.9%, 75 of 95) of patients with peristalsis recovery had type II achalasia. The preoperative proportion of pan-esophageal pressurization was significantly higher in the peristalsis recovery group compared with the non-recovery group. Furthermore, a significant positive correlation was observed between the preoperative proportion of pan-esophageal pressurization and the degree of postoperative peristalsis recovery (*r* = 0.247, *P *< 0.001). There was no significant difference in the proportion of preoperative premature contractions between the groups, which might be attributed to the limited number of type III achalasia cases.

**Table 2:** Clinical characteristics before POEM and myotomy length during the surgery compared between patients with partial recovery of peristalsis and those without recovery.

Variables	Partial recovery of peristalsis (*n *= 95)	No partial recovery of peristalsis (*n *= 195)	*P* value
Male	42 (44.2)	98 (50.3)	0.334
Age, years	42.0 (33.0, 56.0)	42.0 (29.0, 53.0)	0.236
BMI, kg/m^2^	20.6 (18.4, 23.0)	20.3 (18.1, 22.8)	0.403
Duration of symptoms, months	36.0 (12.0, 72.0)	39.0 (22.0, 96.0)	0.591
Preoperative Eckardt score	5.0 (3.0, 7.0)	5.0 (4.0, 7.0)	0.444
Preoperative LES resting pressure, mmHg	35.0 (22.7, 47.3)	33.4 (22.8, 46.1)	0.667
Preoperative IRP, mmHg	22.9 (19.0, 33.3)	24.1 (17.2, 32.2)	0.887
Preoperative proportion of pan-esophageal pressurization	80.0 (30.0, 100.0)	30.0 (0.0, 90.0)	< 0.001
Preoperative achalasia subtypes			< 0.001
Type I	16 (16.8)	82 (42.1)	
Type II	75 (78.9)	110 (56.4)	
Type III	4 (4.2)	3 (1.5)	
Preoperative proportion of premature contractions	0.0 (0.0, 0.0)	0.0 (0.0, 0.0)	0.297
Myotomy length, cm	10.0 (8.0, 11.0)	9.0 (8.0, 10.0)	0.155
Long myotomy	6 (6.4)	8 (4.1)	0.580

Continuous variables are shown as median (interquartile range) and categorical variables are presented as number (percentage).

### Correlation between partial recovery of peristalsis and prognosis

To evaluate the prognosis after POEM, postoperative parameters were compared between the two groups. Despite comparable postoperative manometric parameters, patients who achieved partial peristalsis recovery had significantly better symptom relief, with lower GERDQ scores (although the median and interquartile range were identical between the groups, the overall distributions differed significantly based on the Mann–Whitney *U* test) and a reduced incidence of dysphagia (Table 3). Furthermore, the degree of postoperative peristalsis recovery was significantly negatively correlated with the GERDQ score and dysphagia symptoms (Table 4). These findings suggest that improved peristalsis recovery was associated with enhanced symptom relief.

**Table 3:** Clinical characteristics and manometry parameters after POEM compared between patients with partial recovery of peristalsis and those without recovery.

Variables	Partial recovery of peristalsis (*n *= 95)	No partial recovery of peristalsis (*n *= 195)	*P* value
Postoperative Eckardt score	2.0 (1.0, 3.0)	2.0 (1.0, 3.0)	0.208
Postoperative GERDQ score	6.0 (6.0, 8.0)	6.0 (6.0, 8.0)	0.030
Postoperative LES resting pressure, mmHg	14.2 (7.5, 17.8)	14.2 (9.6, 19.7)	0.344
Postoperative IRP, mmHg	7.2 (3.1, 9.6)	7.8 (3.4, 12.5)	0.291
Postoperative GERD symptoms	19 (20.0)	44 (22.6)	0.559
Postoperative dysphagia symptoms	16 (16.8)	67 (34.4)	0.001
Postoperative reflux esophagitis	31 (32.6)	48 (24.6)	0.181

Continuous variables are shown as median (interquartile range) and categorical variables are presented as number (percentage).

**Table 4:** Spearman correlation analysis between clinical characteristics and manometry parameters and the degree of peristalsis recovery after POEM.

Variables	Total proportion of normal, weak, and fragmented contractions	Postoperative IRP	Postoperative LES resting pressure	Postoperative Eckardt score	Postoperative GERDQ score	Postoperative GERD symptoms	Postoperative dysphagia	Postoperative reflux esophagitis
Total proportion of normal, weak, and fragmented contractions	–	-0.083	-0.059	-0.084	-0.146*	-0.026	-0.164**	0.087
Postoperative IRP	-0.083	–	0.591**	0.084	-0.018	0.050	0.111	-0.075
Postoperative LES resting pressure	-0.059	0.591**	–	0.084	0.077	0.040	0.155*	-0.109
Postoperative Eckardt score	-0.084	0.084	0.084	–	0.369**	0.229**	0.102	-0.093
Postoperative GERDQ score	-0.146*	-0.018	0.077	0.369**	–	0.334**	0.019	-0.017
Postoperative GERD symptoms	-0.026	0.050	0.040	0.229**	0.334**	–	0.118*	0.207**
Postoperative dysphagia	-0.164**	0.111	0.155*	0.102	0.019	0.118*	–	-0.027
Postoperative reflux esophagitis	0.087	-0.075	-0.109	-0.093	-0.017	0.207**	-0.027	–

A dash (–) indicates that the item was not available; *P < 0.05, **P < 0.01, ***P < 0.001.

### Subgroup analysis according to preoperative achalasia subtype

To control for potential confounding factors, patients were stratified into three groups by preoperative achalasia subtype (types I, II, and III). Patients with partial peristalsis recovery showed a significantly higher proportion of preoperative pan-esophageal pressurization in type II achalasia [90.0% (70.0%, 100%) vs 80.0% (50.0%, 100%), *P *= 0.025] and type I achalasia [0.0% (0.0%, 10.0%) vs 0.0% (0.0%, 0.0%), *P *= 0.008], whereas no significant difference was found in type III achalasia, possibly due to the limited sample size. Furthermore, in patients with type II achalasia, those with partial recovery of peristalsis exhibited significantly reduced dysphagia and lower GERDQ scores compared with those without (Table 5). In type I achalasia, patients with peristalsis recovery also showed lower Eckardt scores. Notably, in this subgroup, patients with pan-esophageal pressurization at 10.0% (*n *= 19) exhibited both a significantly higher proportion of peristalsis recovery (36.8% vs 11.4%, *P *= 0.013) and a greater degree of recovery [60.0% (20.0%, 80.0%) vs 0.0% (0.0%, 0.0%), *P *= 0.011] compared with those without pan-esophageal pressurization (*n *= 79).

**Table 5:** Clinical characteristics and manometry parameters after POEM compared between patients with partial recovery of peristalsis and those without recovery in three subtypes of achalasia.

Variables		Partial recovery of peristalsis type I (*n *= 16), type II (*n *= 75), type III (*n *= 4)	No partial recovery of peristalsis type I (*n *= 82), type II (*n *= 110), type III (*n *= 3)	*P* value
Type I achalasia (*n *= 98)	Postoperative Eckardt score	1.0 (0.0, 2.0)	2.0 (1.0, 3.0)	0.022
	Postoperative GERDQ score	6.0 (6.0, 8.0)	6.0 (6.0, 8.0)	0.732
	Postoperative LES resting pressure, mmHg	11.7 (5.3, 22.2)	14.2 (8.6, 19.9)	0.511
	Postoperative IRP, mmHg	3.2 (1.4, 8.6)	7.5 (3.1, 12.7)	0.097
	Postoperative GERD symptoms	6 (37.5)	24 (29.3)	0.555
	Postoperative dysphagia symptoms	5 (31.3)	29 (35.4)	0.727
	Postoperative reflux esophagitis	8 (50.0)	22 (26.8)	0.076
Type II achalasia (*n *= 185)	Postoperative Eckardt score	2.0 (1.0, 3.0)	2.0 (1.0, 3.0)	0.732
	Postoperative GERDQ score	6.0 (6.0, 7.8)	6.0 (6.0, 8.0)	0.042
	Postoperative LES resting pressure, mmHg	11.0 (7.5, 17.6)	14.4 (10.4, 19.6)	0.256
	Postoperative IRP, mmHg	7.2 (3.8, 9.9)	8.3 (3.6, 12.4)	0.342
	Postoperative GERD symptoms	13 (17.3)	19 (17.3)	0.961
	Postoperative dysphagia symptoms	9 (12.0)	37 (33.6)	0.001
	Postoperative reflux esophagitis	22 (29.3)	26 (23.6)	0.426
Type III achalasia (*n *= 7)	Postoperative Eckardt score	2.5 (0.5, 3.0)	3.0 (1.0, –)	0.800
	Postoperative GERDQ score	6.0 (6.0, 8.3)	10.0 (6.0, –)	0.533
	Postoperative LES resting pressure, mmHg	23.7 (13.0, 34.8)	17.3 (7.3, –)	0.400
	Postoperative IRP, mmHg	13.7 (6.1, 23.7)	5.6 (5.5, –)	0.400
	Postoperative GERD symptoms	0 (0.0)	1 (33.3)	0.333
	Postoperative dysphagia symptoms	2 (50.0)	1 (33.3)	>0.999
	Postoperative reflux esophagitis	1 (25.0)	0 (0.0)	>0.999

Continuous variables are shown as median (interquartile range) and categorical variables are presented as number (percentage). A dash (–) indicates that the item was not available.

### Multivariate analysis on the factors associated with peristalsis recovery

To further identify factors independently associated with partial recovery of peristalsis, a multivariate analysis was conducted. After adjustment for age, sex, BMI, disease duration, myotomy length, achalasia subtypes, preoperative and postoperative IRP, and preoperative Eckardt score, the preoperative proportion of pan-esophageal pressurization remained independently associated with peristalsis recovery (odds ratio = 1.02, *P *= 0.007; Table 6).

**Table 6:** Multivariate logistic regression analysis of partial recovery of peristalsis in patients with achalasia.^a^

Variables	Odds ratio (95% confidence interval)	*P* value
Age	1.01 (1.00, 1.03)	0.154
Male	0.85 (0.48, 1.51)	0.585
BMI	1.00 (0.93, 1.08)	0.930
Preoperative Eckardt score	1.00 (0.88, 1.14)	0.994
Achalasia subtypes	–	0.102
Type III (reference)	1.00	–
Type I	0.17 (0.03, 0.89)	0.037
Type II	0.18 (0.03, 1.05)	0.057
Preoperative proportion of pan-esophageal pressurization	1.02 (1.01, 1.03)	0.007
Preoperative IRP	0.99 (0.97, 1.02)	0.656
Postoperative IRP	0.98 (0.95, 1.02)	0.291
Duration of symptoms	1.00 (1.00, 1.00)	0.965
Myotomy length	0.96 (0.84, 1.10)	0.569

a After adjustment for age, sex, BMI, disease duration, myotomy length, achalasia subtypes, preoperative and postoperative IRP, and preoperative Eckardt score.

## Discussion

In this retrospective cohort study, we found that partial recovery of peristalsis was observed in 32.8% of patients with achalasia after POEM, which was associated with preoperative pan-esophageal pressurization. Patients with a return of peristalsis had better relief in GERD and dysphagia symptoms.

Previous studies have explored the potential of peristalsis recovery post-POEM, with recovery rates ranging from 25.0% to 60.9% [14–16]. However, the clinical significance of this recovery remains incompletely understood. Several studies have compared the prognosis of patients with and without peristalsis recovery, yielding inconsistent results. Shi *et al.* reported that patients with partial recovery of esophageal peristalsis had a significantly lower incidence of GERD symptoms and reflux esophagitis [17]. Another study demonstrated that partial peristalsis recovery, rather than the return of IRP, was associated with symptom improvement [18]. However, a study by Vackova *et al.* found no significant impacts of partial peristalsis recovery on the symptomatic outcomes, esophageal emptying assessed by timed barium esophagogram, or Eckardt score [14]. In the present study, patients with partial recovery of esophageal peristalsis had significantly lower GERDQ scores and a reduced incidence of dysphagia symptoms, underscoring the clinical relevance of peristalsis restoration after POEM.

To date, consensus on the factors influencing peristalsis recovery remains elusive. A single-center retrospective cohort study from China reported that 27.3% of patients experienced partial recovery of esophageal peristalsis post-POEM, primarily in those with type II achalasia [17]. Multivariate analysis identified the preoperative LES resting pressure and Eckardt score as independent predictors of peristalsis recovery. Similarly, a multicenter study found that 19.6% of patients (121 of 618) demonstrated post-POEM peristalsis recovery, primarily among type II and type III achalasia cases, with a higher preoperative IRP predictive of recovery [18]. These results suggest a potential association between achalasia subtypes and peristalsis recovery. Nevertheless, Vyas *et al.* found that peristalsis was significantly regained in patients with type I achalasia, while some peristalsis was lost in type III achalasia [19]. Taken together, these observations indicated that the achalasia subtype might not be the primary determinant of peristalsis recovery. In our cohort, a higher preoperative proportion of pan-esophageal pressurization was associated with the postoperative recovery of esophageal peristalsis, particularly in type I and type II achalasia. Furthermore, this association remained significant after adjustment for achalasia subtype in the multivariable model, supporting this hypothesis.

The mechanisms underlying peristalsis recovery remain unclear. Although all subtypes of achalasia are characterized by elevated IRP, they differ in motility patterns. HRM defines type I achalasia by the complete absence of peristalsis, type II by at least two pan-esophageal pressurizations, and type III by at least two premature contractions during 10 supine swallows [3]. These patterns suggest that, while LES relaxation is impaired, the degree of esophageal body dysfunction varies across subtypes. Specifically, the presence of pan-esophageal pressurization in type II and premature contractions in type III may reflect better preservation of esophageal contractile function [20, 21]. Consequently, the relief of LES obstruction may enable these patients to recover peristalsis. This is supported by a positive correlation between the proportion of preoperative pan-esophageal pressurization and the degree of postoperative peristalsis recovery in our study. In type III achalasia, no significant correlation was found between premature contractions and peristalsis recovery, potentially due to the limited sample size.

This study holds significant clinical implications. On the one hand, the preoperative proportion of pan-esophageal pressurization may inform individualized surgical planning and postoperative follow-up strategies for patients with achalasia. On the other, although our previous proteomic analysis of the LES across achalasia subtypes revealed no significant differences in protein expression [22], the present findings indicate that esophageal body contractile activity varies among subtypes, highlighting that future investigations into achalasia pathogenesis should extend beyond the LES to encompass the pathophysiological characteristics of the esophageal body.

There were several limitations in this study. Firstly, the sample size in our study was relatively small, particularly in the group of type III achalasia, which included only seven individuals. In addition, among patients who demonstrated postoperative peristaltic recovery, only a small proportion (*n *= 6) achieved normal peristalsis, while the majority were classified as having ineffective esophageal motility. Therefore, further subgroup analyses according to different patterns of postoperative motility recovery were not feasible. Moreover, postoperative esophageal clearance was not assessed because neither timed barium esophagram nor high-resolution impedance manometry was routinely performed after POEM in our cohort. Furthermore, as this was a retrospective study, the generalizability of our findings was limited. While we established an independent correlation between preoperative pan-esophageal pressurization and postoperative peristalsis recovery, we could not prove a causal relationship. Last but not least, given that current mechanistic studies on achalasia have primarily focused on the LES muscle rather than the esophageal body, further studies on the potential mechanisms of peristalsis recovery post-POEM are warranted.

## Conclusions

A higher preoperative proportion of pan-esophageal pressurization was independently associated with the postoperative recovery of esophageal peristalsis in patients with achalasia. Peristalsis recovery was related to superior relief of dysphagia and GERD symptoms. Consequently, preoperative assessment of pan-esophageal pressurization might help guide individualized postoperative follow-up and patient counseling in patients with achalasia.

## Ethics statements

This study was approved by the Ethical Review Board of the four academic hospitals in China [IRB no. 2022(405); IRB no. (2023)S-No.133; IRB no. I-26PJ0540; IRB no. 2019–212-02]. Written informed consent was obtained from each participant.

## Authors’ contributions

D.J., R.Z., P.D., and S.C. were responsible for the acquisition, analysis, and interpretation of the data, as well as the drafting of the manuscript; N.Z. and Y.X. were responsible for the study concept and the design and analysis of the data, as well as finalizing and approving the manuscript. Other authors were responsible for the acquisition and interpretation of the data. All authors have read and approved the final version of the manuscript.
